# Chat Demeter: a multi-agent system for plant disease diagnosis integrating CNN-transformer models

**DOI:** 10.3389/fpls.2025.1695227

**Published:** 2026-01-19

**Authors:** Sainan Zhang

**Affiliations:** College of Humanities and Law, Zhejiang A&F University, Hangzhou, China

**Keywords:** CNN–transformer models, deep learning, digital agriculture, multi-agent systems, plant disease diagnosis

## Abstract

Plant diseases remain a significant challenge in global agricultural production. Achieving efficient and accurate disease detection is essential for reducing crop losses, controlling agricultural costs, and improving yields. As agriculture rapidly advances toward digitalization and intelligent transformation, the application of artificial intelligence technologies has become a key pathway to enhancing industrial competitiveness. In this study, Chat Demeter, a multi-agent system for plant disease diagnosis based on deep learning. The system captures real-time leaf images through camera devices. It employs a CNN-Transformer model to perform instance segmentation and object detection, thereby enabling automatic identification of diseased leaves and classification of disease types. To enhance interactivity and practical value, the system incorporates a natural language interface, allowing users to upload images and receive automated diagnostic results and treatment suggestions. Experimental results demonstrate that the system achieves an accuracy of 99.50% and an AUC o**f** 99.91% on the validation dataset, highlighting its superior performance. Overall, Chat Demeter provides an effective tool for crop health monitoring and disease intervention, while offering a feasible pathway and developmental direction for integrating and optimizing future agricultural multi-agent systems.

## 1 Introduction

Agricultural productivity is crucial for economic growth and food security. However, the inability to identify and control plant diseases—especially foliar diseases—can lead to rapid epidemics, causing severe yield losses and economic damage (Shivaprasad and Wadhawan, 2023). Traditionally, disease identification has relied on expert diagnoses or manual visual inspection, methods that are inefficient and impractical for large-scale farming (Yani et al., 2024). Additionally, the limited availability of plant pathology experts often results in delayed disease detection until significant damage has already occurred (Wang et al., 2024). Thus, automated, accurate, and user-friendly methods for early disease detection are essential.

Digital tools are playing an increasingly important role in agriculture. Even in resource-limited regions, the widespread use of smartphones and mobile internet has enabled initiatives such as “Agriculture 4.0” and “data-driven farming” (Mehrabi et al., 2021). Technologies like voice messaging can deliver real-time weather updates and cultivation advice (Cole and Fernando, 2021), while mobile apps assist with spatiotemporal recommendations for fertilizer application (Zossou et al., 2021). The development of diagnostic applications, such as Petrelis’ leaf disease app, has improved accessibility for disease detection in agricultural practice (El Sakka et al., 2025). Consequently, digital agriculture is gaining global attention, with various organizations, governments, and investors promoting digital technology deployment to tackle agricultural challenges (Steinke et al., 2022). Concurrently, advances in computer vision and deep learning are significantly improving disease diagnosis accuracy. Convolutional neural networks (CNNs) have been extensively used for leaf disease classification, achieving over 95% accuracy on benchmark datasets like PlantVillage through transfer learning (Amritraj et al., 2023; Peyal et al., 2023).

Despite these advancements, many state-of-the-art models remain confined to laboratory settings, lacking user-friendly interfaces that limit practical deployment. Mobile apps partially address this issue by enabling AI-powered diagnostic tools to reach farmers more easily (Aryan et al., 2025). However, agent-based technologies offer additional advantages in agricultural contexts. Intelligent agents can autonomously make decisions, optimize performance, and adapt to dynamic environments, making them ideal for real-world farming scenarios (Acharya et al., 2025). By utilizing natural interaction, machine learning, and multi-agent coordination, these systems can perform tasks efficiently while enhancing user engagement (Singh et al., 2024; Hosseini and Seilani, 2025). Thus, integrating agent technology into plant disease diagnosis has the potential to advance agricultural digitalization and improve disease management.

This study proposes Chat Demeter, a multi-agent system for plant disease detection and severity quantification, which combines deep learning-based leaf disease classification with traditional image processing methods. The system accurately identifies healthy and diseased leaves, quantifies disease severity by evaluating lesion areas relative to the total leaf area, and operates in real time with autonomous decision-making capabilities. Implemented with a lightweight deep learning model on the PyTorch framework, Chat Demeter balances accuracy, generalization, and real-time reliability in diverse agricultural environments. The system is integrated into an agent-based framework on the Coze platform, enabling automated recognition and response, reducing the need for human intervention and enhancing deployment flexibility. Ultimately, Chat Demeter provides a user-friendly, interactive platform for farmers to rapidly identify diseases and assess severity, offering a feasible path toward precision disease management in smart agriculture.

### 1.1 Related work

#### 1.1.1 Review of plant pest and disease detection based on traditional image processing methods

Plant pest and disease image recognition has long been essential to intelligent agricultural management. Traditional image processing techniques have a relatively long application history in this field. Early studies typically combined image preprocessing, handcrafted feature extraction, and classical classification algorithms to accomplish disease identification and classification tasks (Liu and Wang, 2021). During training, images usually underwent denoising, enhancement, and background removal to highlight lesion areas and improve classification accuracy (Arivazhagan et al., 2013). Subsequently, researchers applied techniques such as color space conversion, thresholding, and clustering to segment images and extract representative features including shape, color, and texture (Nisar et al., 2020; Ahmad Loti et al., 2021; Chakraborty et al., 2021). These features were the (Domingues et al., 2022; Shoaib et al., 2023) development of artificial intelligence, deep learning—particularly convolutional neural networks (CNNs)—has significantly advanced automation and accuracy in plant pest and disease recognition (Shoaib et al., 2025), Compared with traditional methods, deep learning models possess end-to-end learning capabilities that allow them to automatically extract multi-level discriminative features directly from raw images, thereby substantially reducing the need for manual intervention. Early studies predominantly adopted simple CNN architectures for disease classification. However, they later evolved into more complex deep networks such as ResNet, DenseNet, and InceptionNet, achieving classification accuracies above 95% on benchmark datasets such as PlantVillage (Belmir et al., 2023; Biswas and Yadav, 2023). Moreover, adopting transfer learning has effectively alleviated the problem of limited agricultural image datasets, enhancing both generalization and robustness. High recognition performance can be achieved even with limited labeled samples by pretraining models on large-scale general datasets and subsequently fine-tuning them on agricultural images.

Despite these promising results, deep learning methods still face substantial challenges in real-world applications. Insufficient training data, disease diversity, illumination variations, and overlapping leaves compromise model stability (Mezenner et al., 2023). Furthermore, most models remain confined to research environments and lack seamless integration with user-friendly front-end systems, limiting their accessibility to farmers and practitioners. Therefore, future research must focus on developing more efficient, scalable, and adaptive solutions by integrating deep learning models with intelligent data acquisition systems, mobile applications, and web-based visualization platforms. Such integration will enable the transition of plant disease detection from “high-performance” to “high-usability,” ultimately providing intelligent support for advancing precision agriculture.

### 1.2 Limitations of existing research methods

Although significant progress has been made in combining deep learning with traditional image processing techniques for plant disease detection, several limitations remain. These limitations include: (1) model generalization being constrained by the limited scale and diversity of available datasets; (2) the lack of efficient intelligent decision-making mechanisms, which results in insufficient system autonomy; and (3) existing technologies struggling to simultaneously satisfy the dual requirements of real-time performance and high accuracy under complex field conditions. To address these issues, this study introduces a novel framework that integrates intelligent agent technology with state-of-the-art CNN-based architectures for real-time monitoring and management of plant diseases. This approach not only mitigates the challenges of resource limitations and scalability but also enhances system intelligence and autonomy. In recent years, agent-based collaborative systems have demonstrated significant advantages in protein analysis and medical image diagnosis domains. AI agents are capable of performing complex tasks, planning and optimizing workflows, and conducting self-assessment to identify and bridge knowledge gaps, thereby improving decision accuracy, system flexibility, and autonomy (Gao et al., 2024), For instance, recent work has integrated exosome Raman spectroscopy with large language models to develop intelligent diagnostic agents, achieving efficient, interpretable recognition and clinical decision support (Yang et al., 2025). These experiences provide valuable insights for agriculture, suggesting that the fusion of intelligent agents and deep learning can establish adaptive collaborative systems tailored for agricultural applications, effectively addressing current plant disease detection systems’ shortcomings in intelligence and generalizability.

### 1.3 Research contributions

The contributions of this study are primarily reflected in two aspects:

#### 1.3.1 Systematic evaluation of deep learning architectures

Six state-of-the-art deep learning models (CSPNet, HRNet, GhostNet, ResNet50, ViT, and GSDCNet) were systematically evaluated for rice pest and disease image recognition. Their performance was compared across multiple dimensions, including accuracy, inference speed, and model complexity, to identify the optimal architecture comprehensively.

#### 1.3.2 Development of an agent-based prototype system

Leveraging the best-performing model, we developed a prototype intelligent agent system tailored for real-world agricultural scenarios. This system provides high recognition accuracy and real-time feedback, meeting the practical demands of field-level pest and disease diagnosis. A dataset covering representative leaf diseases and pests was used for training and validation, enhancing model robustness and generalizability, and supporting innovative agriculture applications.

## 2 Materials and methods

**Figure 1:**
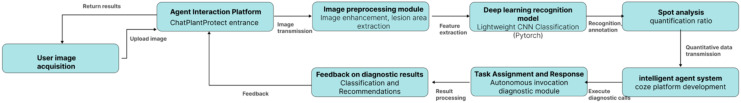
Overall workflow.

Convolutional neural networks (CNNs) have been widely applied in recognizing and diagnosing plant diseases. However, most existing studies have focused on disease category classification, while paying insufficient attention to the fine-grained identification of different infection stages. Therefore, the main objective of this study is to design and develop an integrated solution that combines intelligent agent technology with deep learning-based classification to enable real-time and accurate Detection of plant disease infections (see **Figure 1**).

**Figure 2:**
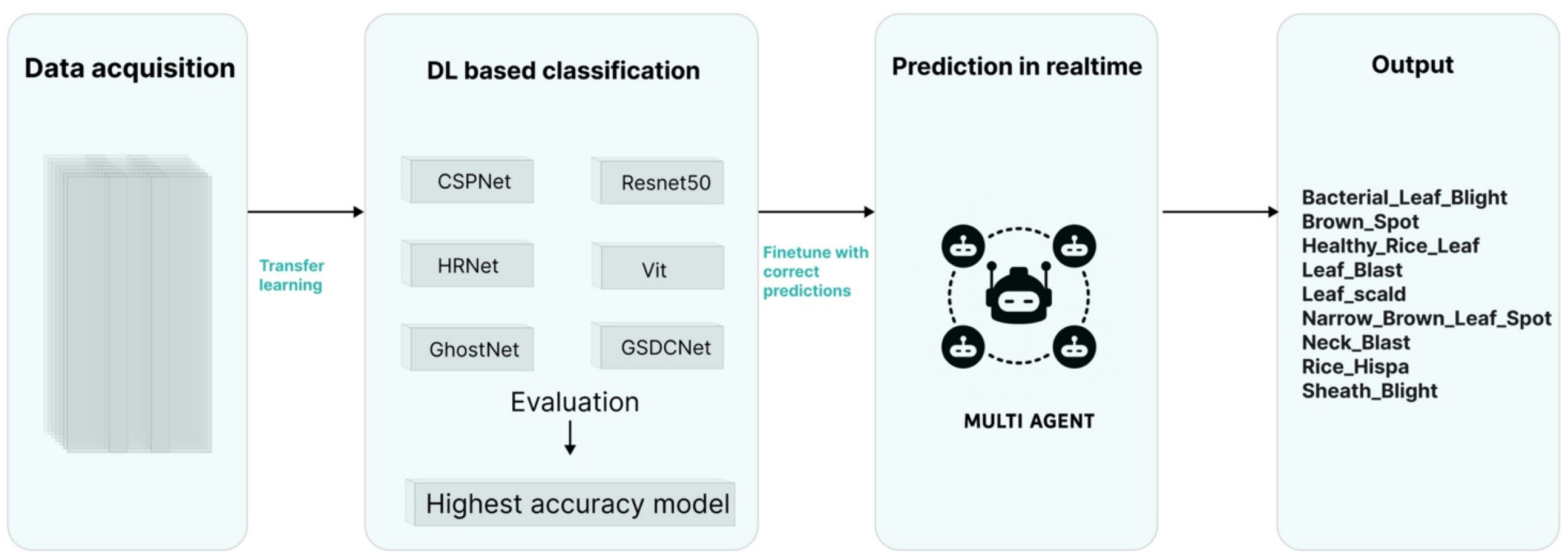
Overall architecture of a multi-agent system.

First, the acquired image dataset was subjected to basic preprocessing to meet the requirements of subsequent model training. The preprocessed dataset was then used to train five state-of-the-art CNN models, including CSPNet, HRNet, GhostNet, ResNet50, and ViT. By comparing and analyzing model performance, the optimal model was identified and subsequently integrated into the intelligent agent system, enabling automated real-time Detection of disease infection stages and providing practical support for agricultural management decisions. **Figure 2**. illustrates the overall architecture of the proposed solution and its technical roadmap. The system framework consists of four core modules: a plant disease image acquisition and preprocessing module, a deep learning-based detection module, a multi-agent interaction module, and a visualization module. The technical roadmap encompasses the end-to-end process from data acquisition and model training to practical deployment. Within the multi-agent interaction architecture, four types of agents are defined—task planning, inference, evaluation, and visualization—responsible for task decomposition and decision-making, deep learning-based detection, performance evaluation, and user interaction, respectively. This design establishes a scalable and intelligent platform for plant disease diagnosis.

### 2.1 Data acquisition and preprocessing

To develop a robust plant pest and disease recognition model with strong generalization capability, the image dataset used in this study was primarily obtained from the public database Kaggle (https://www.kaggle.com/), a widely recognized platform for data science and machine learning research. As a prominent global community, Kaggle provides abundant resources to support various research activities. The dataset employed in this work covers multiple common disease and pest categories and was utilized for model construction and training. The dataset was carefully organized and systematically preprocessed to ensure training efficiency and accuracy, laying a solid foundation for subsequent model optimization. A detailed list of the nine disease categories relevant to diagnostic decision-making is provided in **Supplementary Table 1**. The original dataset can be accessed at https://www.kaggle.com/code/anshulm257/rice-disease-detection-using-cnn, which includes four distinct datasets to ensure diversity in data sources: https://www.kaggle.com/datasets/nirmalsankalana/rice-leaf-disease-image, https://www.kaggle.com/datasets/jay7080dev/rice-plant-diseases-dataset; https://www.kaggle.com/datasets/loki4514/rice-leaf-diseases-detection.

Based on the original dataset, preprocessing operations were performed to improve image quality and enhance the model’s adaptability to complex agricultural environments. First, all images were resized to a uniform input dimension of 224 × 224 pixels to match the input requirements of deep learning models. Second, pixel values were normalized to the range (0,1), accelerating model convergence and improving training stability. Since field images are often affected by illumination variation and leaf occlusion, data augmentation strategies were further applied, including random rotations (± 15°), horizontal flipping, and brightness/contrast perturbations, to simulate diverse real-world inputs and enhance model robustness and generalization. Additionally, to reduce background noise interference, HSV color space segmentation and Otsu’s thresholding were employed for partial background removal, highlighting the salient lesion regions and strengthening the model’s attention to critical areas.

### 2.2 Model development and evaluation

In this study, we adopt a hybrid deep learning architecture that integrates convolutional neural networks (CNNs) with Transformer structures, leveraging their complementary strengths in feature extraction and generalization. The proposed model comprises key components, including convolutional layers, attention mechanisms, and classification heads. Data augmentation, transfer learning, and hyperparameter optimization (e.g., learning rate adjustment and batch size tuning) were employed to enhance performance.

The model design was guided by a balance of accuracy, computational cost, and generalization capability, with the ultimate goal of achieving precise Detection of plant pest and disease images. Conventional CNNs demonstrate strong capability in local feature extraction but are limited by restricted receptive fields and insufficient modeling of long-range dependencies. Conversely, Transformer-based models excel in capturing global features and modeling long-range relationships, yet are prone to overfitting when trained on small-scale datasets.

**Figure 3:**
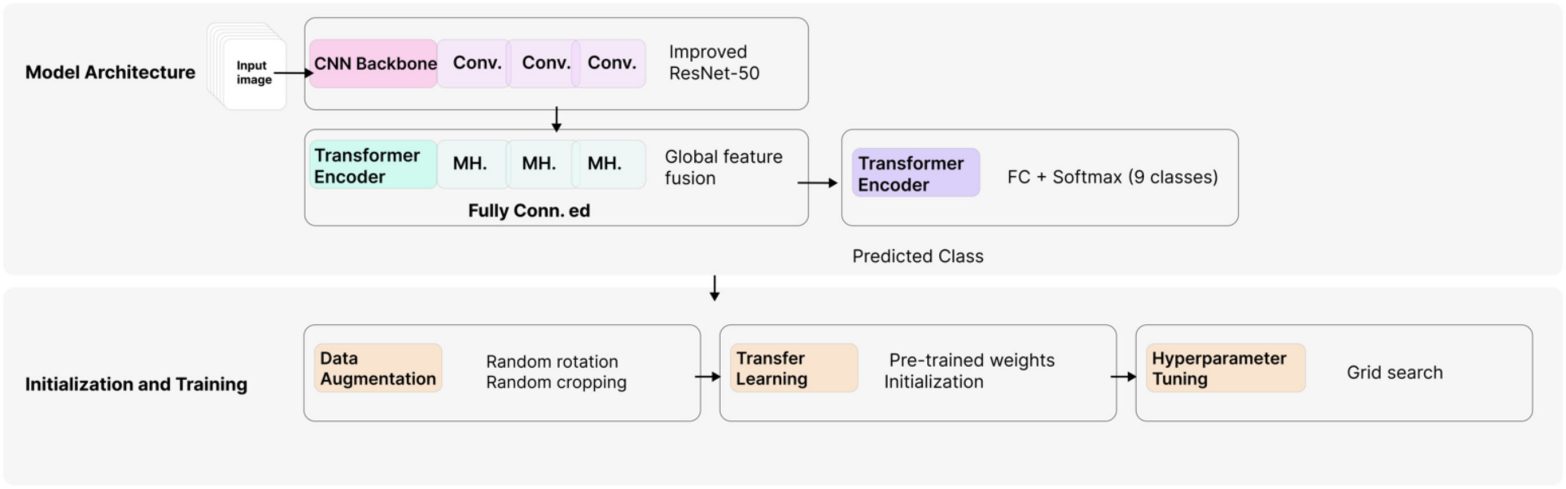
Overall structure of the model.

To overcome these limitations, we propose a CNN-Transformer hybrid architecture that effectively combines CNNs’ ability to capture fine-grained local features with Transformers’ strength in global dependency modeling. This integration enables a comprehensive representation of local and global image features, thereby improving classification accuracy and model generalization. The proposed hybrid architecture ultimately provides a robust and practical plant pest and disease detection solution (see **Figure 3**).

#### 2.2.1 Model architecture and parameter settings

##### 2.2.1.1 Feature extraction module (CNN backbone)

An improved ResNet-50 architecture was employed as the backbone for feature extraction. The network efficiently captured fine-grained local features of diseased leaf images through four main convolutional blocks, each configured with a kernel size of 3×3, a stride of 1, and *same padding*. Batch Normalization and ReLU activation functions were applied to each convolutional layer to improve training stability and alleviate the vanishing gradient problem.

##### 2.2.1.2 Feature fusion module (transformer encoder)

A Transformer encoder was introduced on top of the CNN-extracted spatial features to further exploit global contextual information. This module consisted of four Transformer encoder layers, each incorporating eight multi-head self-attention mechanisms with an embedding dimension of 512. Layer Normalization was applied to ensure stable feature distribution, enhancing generalization ability and convergence efficiency.

##### 2.2.1.3 Classification module (classification head)

For final disease classification, a fully connected layer followed by a Softmax function was used to map the fused feature vectors to the target classes. The number of neurons in the fully connected layer corresponded to the number of categories in the dataset, while Softmax activation produced probability distributions for multiclass prediction.

#### 2.2.2 Model initialization and training

##### 2.2.2.1 Data augmentation

Multiple augmentation strategies, including random rotations, horizontal flipping, random cropping, and color jittering, were applied to mitigate overfitting under limited training data. These operations expanded the adequate dataset size and enhanced robustness to real-world variations in illumination and occlusion.

##### 2.2.2.2 Transfer learning

The CNN backbone was initialized with weights pretrained on the large-scale ImageNet dataset. This general visual feature knowledge transfer accelerated convergence and reduced the risk of overfitting.

##### 2.2.2.3 Hyperparameter tuning

Optimal hyperparameters were identified through a grid search strategy. The initial learning rate was set to 1e-4 and dynamically adjusted using a Cosine Annealing scheduler. A batch size of 32 was adopted, and additional optimizer parameters were fine-tuned during training to maximize model performance.

#### 2.2.3 Model evaluation

Multiple evaluation metrics, including accuracy, recall, precision, F1-score, and AUC, were employed to assess model performance comprehensively. The best-performing model was selected and integrated into the intelligent agent system based on these evaluation results.

### 2.3 Multi-agent system

**Figure 4:**
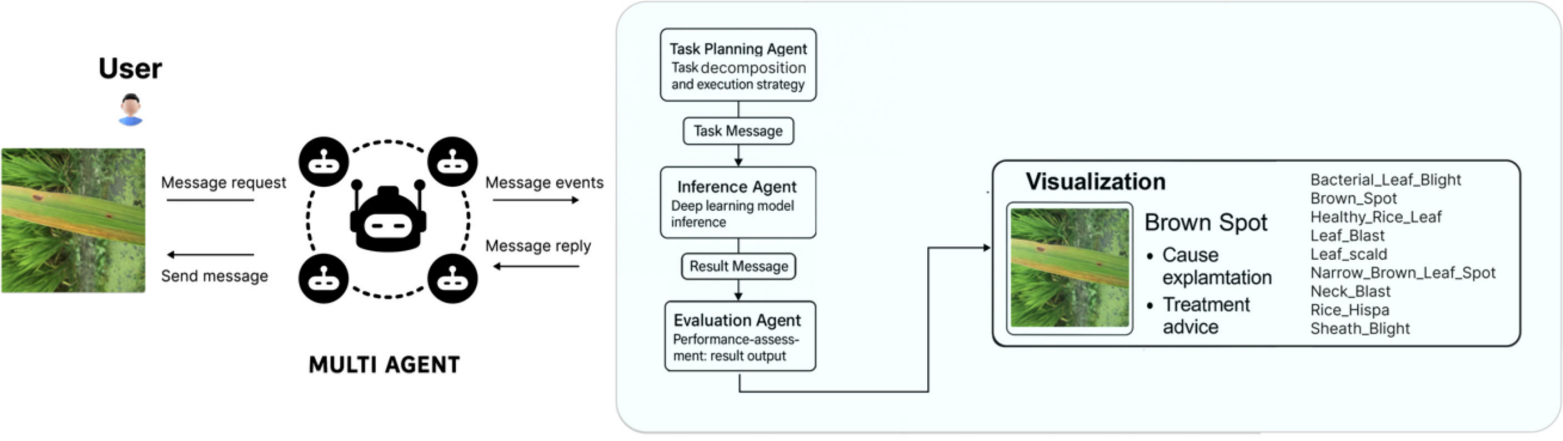
Integrated agent structure diagram.

The proposed multi-agent system (MAS) consists of four functionally independent and clearly defined agents: Task Planning Agent, Inference Agent, Evaluation Agent, and Visualization Agent. These agents collaborate through well-defined message-passing and data exchange protocols, improving system autonomy, operational efficiency, and detection accuracy (see **Figure 4**).

#### 2.3.1 Task planning agent

The Task Planning Agent is the entry point of system interaction, responsible for parsing user commands and formulating execution strategies. Its functions include:

Receiving user detection requests and analyzing task requirements, including target objects, disease categories, real-time constraints, and environmental parameters; Designing optimal task execution strategies using heuristic algorithms and decision-tree models, while considering real-time system status, ensuring resource utilization above 85%; Generating task messages and metadata (e.g., image dimensions, number of samples, task priority) and coordinating the Inference Agent’s execution order and resource allocation, keeping task scheduling latency within 50 ms.

#### 2.3.2 Inference agent

The Inference Agent is responsible for disease classification and Detection. Its functions include:

Receiving image detection tasks from the Task Planning Agent. Performing feature extraction and classification using the CNN-Transformer hybrid model (introduced in Section 3.1), and outputting predicted categories with confidence scores in real time; Ensuring classification accuracy of at least 93%, with inference latency maintained within 100 ms; Packaging detection results and metadata for transmission to the Evaluation Agent for further analysis.

#### 2.3.3 Visualization agent and collaboration mechanism

The Visualization Agent provides users an interactive interface for intuitive result presentation and analysis. Its functions include:

Integrating detection results from the Inference Agent and performance metrics from the Evaluation Agent in real time; Employing interactive charts and heatmaps to visually present disease categories, confidence scores, detection distributions, and performance indicators, with presentation accuracy exceeding 98%;

Supporting interactive analysis, including real-time queries, historical trend analysis, and user feedback, enhances user understanding and trust in the detection process. Agent communication and collaboration are implemented using a message-passing mechanism, with the following core message types:

Task Message: issued by the Task Planning Agent to the Inference Agent, containing task instructions, data sources, and parameters; Result Message: sent from the Inference Agent to the Evaluation Agent, including classification results, confidence scores, and inference latency; Evaluation Feedback Message: The evaluation agent returned this message to the task planning agent, who provided performance metrics and optimization suggestions. Message exchange follows a lightweight request–response protocol based on JSON format, enabling efficient deployment in real-world agricultural scenarios.

### 2.4 Experimental environment and setup

This study’s model training and evaluation were conducted on a high-performance server equipped with an NVIDIA Tesla V100 GPU, featuring 5,120 CUDA cores and 16 GB of HBM2 memory, providing substantial computational support for large-scale deep learning tasks. Due to its stability, reliability, and strong open-source community support, the server operated on Ubuntu 18.04 LTS, a widely adopted operating system in big data and machine learning research. TensorFlow 2.4 was chosen as the primary deep learning framework, offering flexible model design capabilities and efficient GPU utilization. NumPy and Pandas libraries were employed for data preprocessing and analysis, while the h5py library was used for model serialization and storage.

The dataset used in this study consisted of 3,172 diseased leaf images, covering nine common crop disease categories, with approximately 700 images per class. The dataset was split into a 70% training set (2,220 images), a 15% validation set (476 images), and a 15% test set (476 images) for model training, hyperparameter tuning, and final performance evaluation.

## 3 Result

### 3.1 Classification results

Multiple performance metrics were employed for systematic assessment to evaluate the effectiveness of the proposed model in diseased leaf diagnosis. As presented in **Table 1**, the CNN–Transformer hybrid model consistently outperformed both conventional CNN models and standalone Transformer architectures, achieving superior accuracy and generalization capability and thereby demonstrating stronger classification performance and robustness.

**Table 1:** Model performance metrics.

Model	Acc	Recall	f1-score	Precision	Parameters
CSPNet	0.9855	0.9865	0.9857	0.9852	21616168
HRNet	0.983	0.9832	0.9831	0.983	19254102
GhostNet	0.9898	0.9904	0.99	0.9897	3905351
ResNet50	0.9821	0.9822	0.9819	0.9823	23526473
Vit	0.9574	0.9592	0.9586	0.9583	85800794
**GSDCNet**	**0.9991**	**0.9994**	**0.9992**	**0.9991**	**2723349**

In evaluating mainstream pretrained models, performance was compared using accuracy, recall, precision, F1-score, and confusion matrix analysis. Among the tested models, GSDCNet demonstrated the best overall performance, achieving an accuracy of 99% while maintaining balanced precision, recall, and F1-scores across all categories. Notably, the model attained a recall of 99.94% and an F1-score of 99.92%, highlighting its superior capability (as shown in the bold text of **Table 1**).

**Figure 5:**
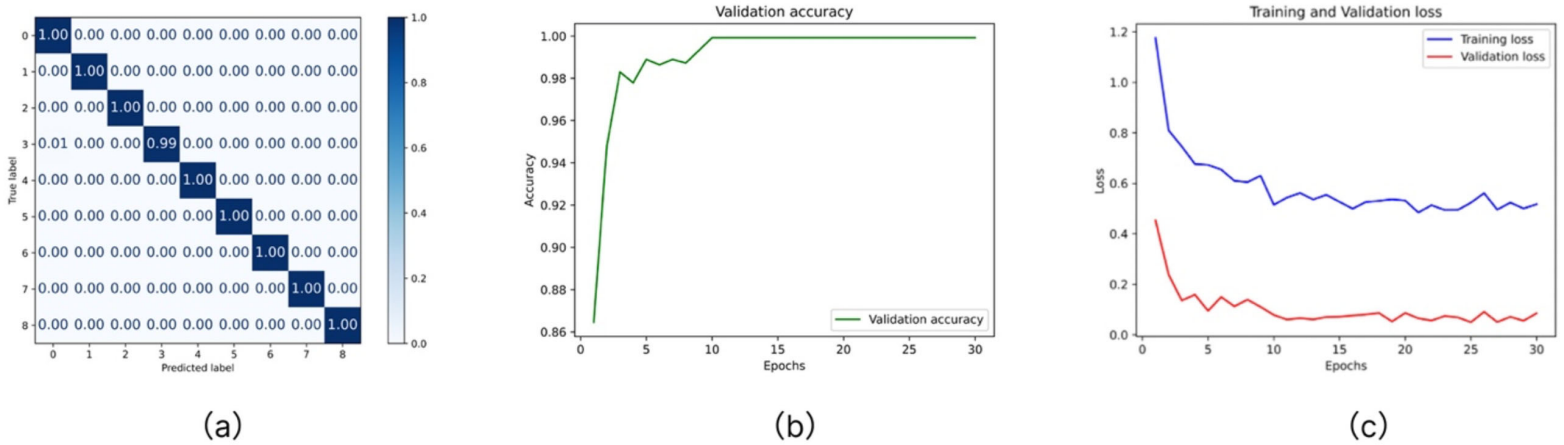
Model performance results (**a**. Confusion matrix **b**. Validation accuracy **c**. Training and Validation Loss).

**Figure 5**. provides a visualization of three key performance aspects. **Figure 5A** shows that the normalized confusion matrix illustrates excellent classification performance across nine disease categories, with all diagonal values exceeding 0.99, indicating high precision and recall. **Figure 5B** depicts the validation accuracy curve, which surpassed 98% within the first five epochs and converged to nearly 100% after the tenth epoch. **Figure5C** shows the training and validation loss curves, which followed a steadily decreasing trend. The validation loss consistently remained below the training loss without significant fluctuations or signs of overfitting, suggesting stable training dynamics and good convergence properties.

### 3.2 Model integration in the agent-based application

Given its superior overall performance, the GSDCNet model was converted into the LITERT format and integrated into the Coze platform to construct a workflow for plant pest and disease recognition. Coze supports two types of plugins: local plugins and external service plugins. However, due to the limited computational resources of the local environment, Coze alone was insufficient for handling complex agricultural image processing tasks. To address this limitation, we developed external plugins using the Flask framework, enabling communication between Coze and the plugins via the HTTP protocol. These plugins were designed to provide API-based services to Coze, thereby ensuring high customizability and scalability of the system. This strategy allowed the plugin functionalities to be precisely tailored to the specific requirements of the agricultural diagnostic workflow, thereby enhancing the practicality and adaptability of the large language model (LLM) system in intelligent agricultural recognition.

Users initiate a detection request through the visualization interface during the image detection and agent interaction process. The Task Planning Agent decomposes the request and dispatches tasks; the Inference Agent processes the input image and generates the disease classification results; the Evaluation Agent provides real-time performance metrics and optimization feedback; and finally, the Visualization Agent presents the results through intuitive displays, allowing users to query, analyze, and interpret the outputs.

**Figure 6:**
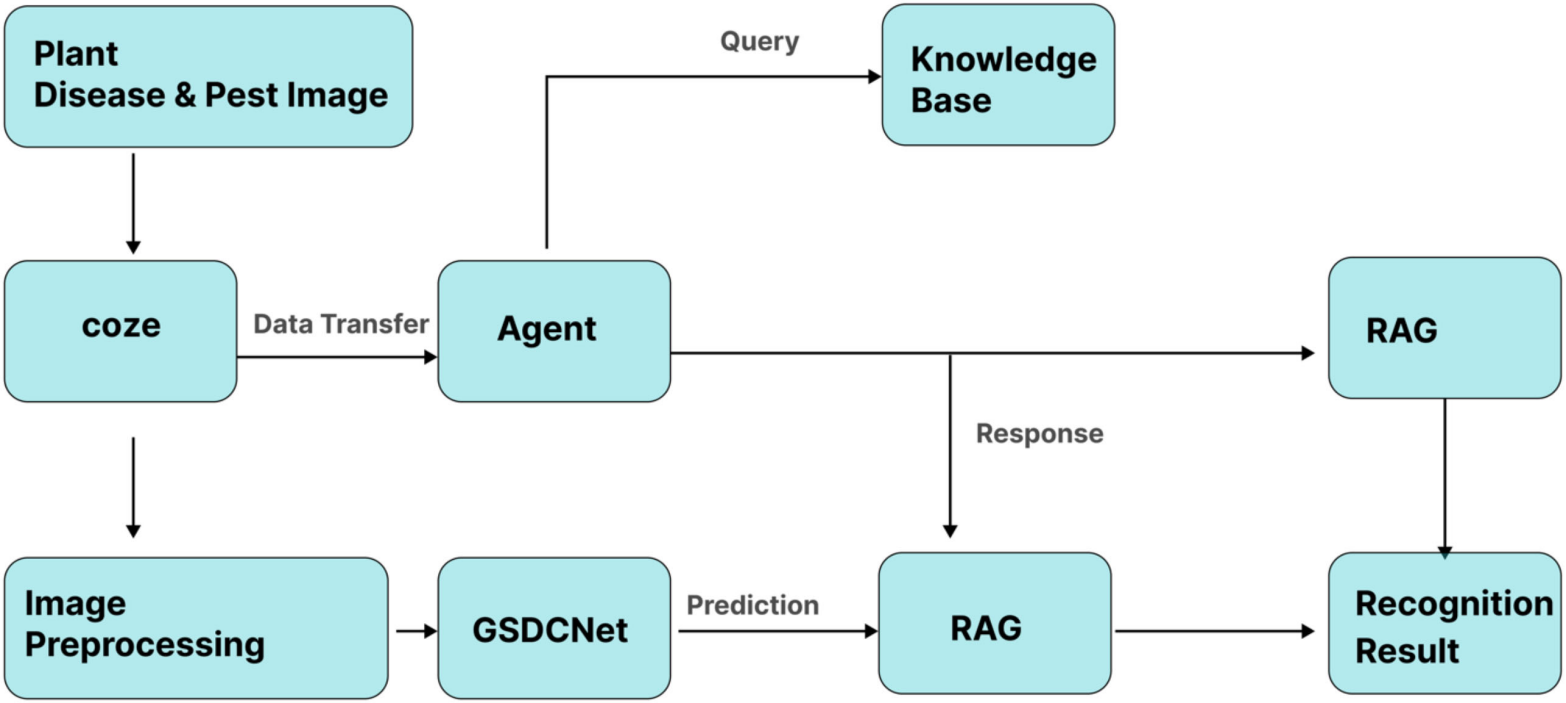
Energy-body interaction path.

The user initiates a detection request through the visualization interface during the image detection and agent interaction process. The Task Planning Agent receives and parses the request, performing task decomposition and dispatch. The Inference Agent then processes the input image, extracting features and generating disease classification results with associated confidence scores. Subsequently, the Evaluation Agent provides real-time feedback on performance metrics and optimization suggestions. Finally, the Visualization Agent integrates the detection results and evaluation outputs, presenting them to the user intuitively and interactively for result querying and analysis (see **Figure 6**).

### 3.3 Visualization of plant disease detection results on the Coze platform

**Figure 7:**
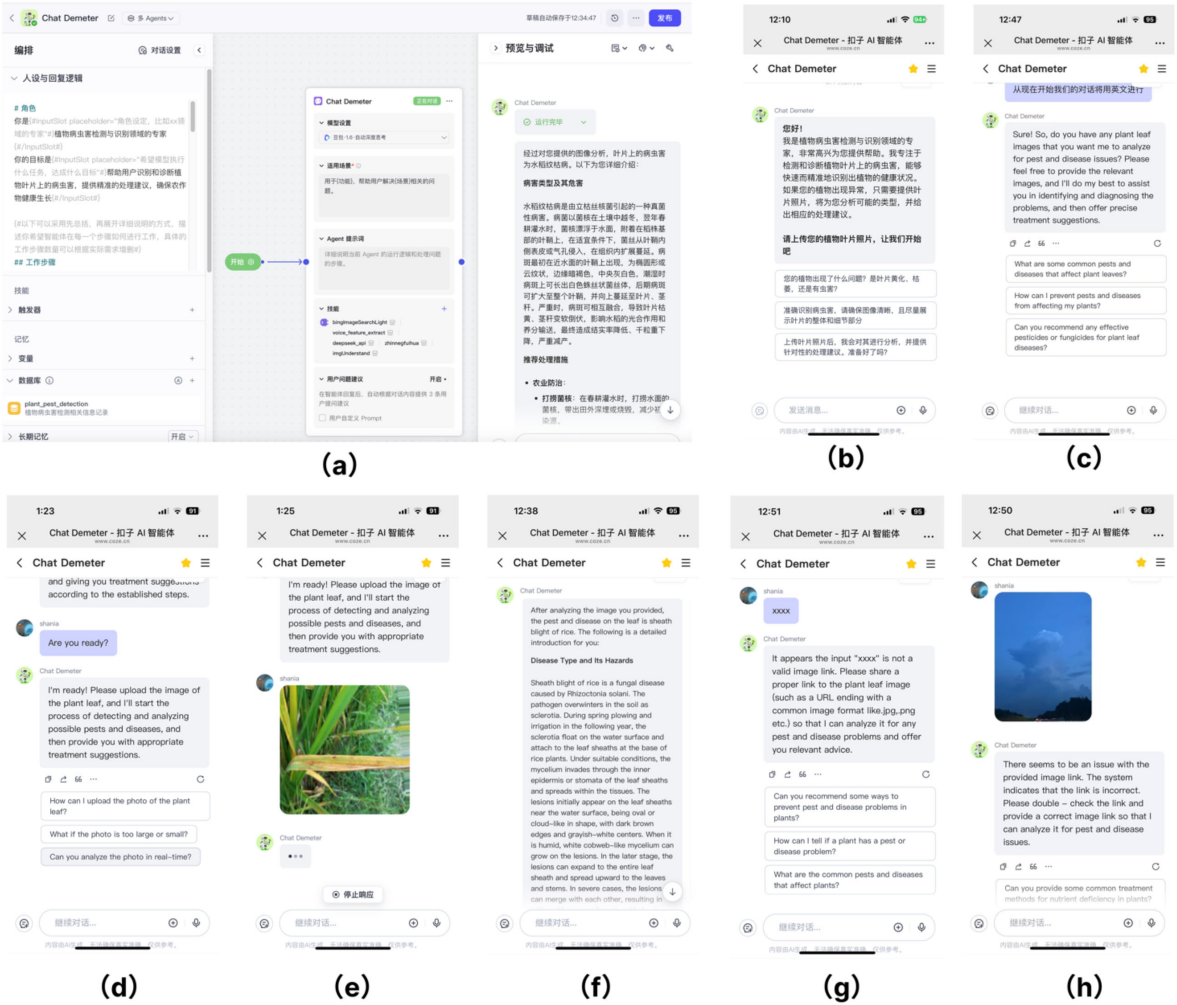
Multi-agent Chat Demeter visual interface. **(a)** The backend logic preset for disease diagnosis utilizing the RAG mechanism. **(b)** The initial interface of the agent's main page. **(c)** Demonstration of switching to English mode. **(d)** Real-time response and guidance based on dialogue context. **(e)** Interactive Q&A regarding plant disease examples. **(f)** Generation of interpretable output results. **(g)** System feedback upon submission of irrelevant text or symbols. **(h)** Guidance on the correct input path when an incorrect image is uploaded.

With the support of the Coze platform (Coze documentation), users can rapidly access plant disease detection results, thereby significantly enhancing user experience and decision-making efficiency. Each plugin interacts with Coze through two components: input and output. For instance, in the case of plant leaf image classification, once Coze identifies the user’s intention to recognize diseases in the image, it converts the image file into either a URL or a base64-encoded string. It forwards it to the appropriate image recognition plugin. The plugin, which integrates a pretrained deep learning classification model, performs inference on the input image and returns the predicted results as output to Coze (**Figure 7**). The system accurately identifies whether the input image exhibits characteristic disease symptoms, such as rice brown streak disease. Additionally, an image similarity mechanism is employed to filter out samples inconsistent with the training distribution; if the similarity score is low, the system flags the image as potentially belonging to an unknown disease class. All image data and models are sourced from publicly available agricultural research datasets.

When the system detects that the user-submitted image is related to a specific crop disease, it automatically invokes the corresponding pretrained model for analysis. It returns precise classification results, demonstrating strong system scalability. Furthermore, Chat Demeter incorporates a retrieval-augmented generation (RAG) mechanism, dynamically integrating reliable agricultural knowledge from external databases, scientific literature, and extension manuals into the conversational context. This approach substantially enhances the domain-specificity and interpretability of generated outputs (**Figures 7A–D**. The system first retrieves relevant knowledge passages on crop disease diagnosis and symptom recognition on mobile devices, combines them with the original query, and passes them to the generation model, which outputs accurate and contextually appropriate responses.

The system also supports multilingual interaction; when users switch to English, the agents seamlessly engage in English dialogue (**Figures 7B, C**). **Figures 7E, F** demonstrate plant disease–related Q&A examples, while **Figures 7G** And seven h show that when irrelevant or incorrect images are submitted, the system provides immediate feedback, alerting users and suggesting the correct input path. This real-time feedback mechanism lets users quickly identify errors and make informed decisions.

The application supports immediate upload and offline storage for data submission, making it adaptable to regions with limited connectivity. The system was extensively tested across iOS and Android to ensure compatibility and efficiency. The results confirmed robust real-time prediction and interaction performance on modern smartphones and tablets, though performance may vary slightly on older or lower-end devices.

## 4 Discussion

This study systematically evaluated five state-of-the-art CNN architectures, with SDCNet achieving the best overall performance. When integrated with a user-friendly visualization agent, the model demonstrated strong applicability in resource-constrained environments, enabling real-time mobile access and continuous dataset expansion through field usage. This adaptability is crucial for long-term improvements in accuracy and robustness, making the system highly valuable in real-world agricultural settings. However, challenges remain that need to be addressed for effective real-world deployment.

The proposed CNN-Transformer hybrid model (CNN-Trans) effectively combines the strengths of CNNs and Transformers, achieving superior accuracy, generalization, and computational efficiency compared to conventional CNNs or standalone Transformer models. With an accuracy of 99.1% on the test set, it outperforms current mainstream methods (Xu et al., 2024; Shi et al., 2025). This performance is promising for practical applications, but real-world scenarios introduce additional constraints that can affect the system’s effectiveness.

A multi-agent collaboration framework was designed at the system level, integrating task planning, inference, evaluation, and visualization agents into a closed-loop workflow. This design supports task scheduling, model inference, performance assessment, and interactive result presentation, overcoming the limitations of single-model approaches. It significantly enhances automation and intelligence in plant disease detection, providing a new paradigm for agricultural diagnostic systems. While the system holds strong potential for real-world applications—such as reducing labor costs, enabling precision pesticide use, ensuring ecological safety, and supporting sustainable agricultural development—several real-world constraints must be carefully considered.

First, the generalization capability of deep learning models in agricultural environments remains limited. Factors such as lighting variations, weather conditions, and leaf occlusion often degrade performance (Ejaz et al., 2025). The current version of the system primarily recognizes specific disease classes labeled in the training dataset, which represent known biotic stresses like fungal, bacterial, or viral diseases. However, it is not explicitly trained to differentiate between biotic and abiotic stresses, such as nutrient deficiencies, environmental stressors (e.g., drought or salinity), or mechanical damage. Expanding the training dataset to include a broader range of stress factors could improve the model’s ability to distinguish between biotic and abiotic causes, addressing one of the key limitations for real-world applicability.

Moreover, overlapping symptoms in plant diseases pose a significant challenge to the accuracy of disease classification. The CNN-Transformer hybrid model helps reduce the risk of misclassification by capturing both local and global features in images, while confidence scores assess the certainty of predictions. Although this helps identify potential misclassification risks, improving model robustness requires diverse data collection, more enhanced augmentation strategies, and the integration of semi-supervised or active learning techniques. These approaches can help the system handle real-world complexity and increase its ability to adapt to new, unforeseen conditions.

Communication delays or failures in the multi-agent system could compromise its reliability and stability. The current lightweight communication protocol enables fast execution of task decomposition, inference, and evaluation, but further optimization of communication protocols, task allocation strategies, and fault-tolerant mechanisms—such as message retransmission, agent state monitoring, anomaly detection, and self-recovery functions—are needed. Addressing these communication constraints will ensure smoother system operation in field environments where network instability may occur. Another practical challenge involves the cost and availability of annotated data. High-quality labeled data are essential for training deep learning models, yet collecting such data can be resource-intensive. Future work could combine expert annotations with molecular confirmation (e.g., PCR) to ensure more accurate ground truth creation. Additionally, techniques such as transfer learning, synthetic data generation using Generative Adversarial Networks (GANs), and domain adaptation methods could help mitigate data scarcity and improve the model’s robustness and generalizability in diverse environments. Finally, while the current system focuses on disease recognition and severity assessment, it does not yet predict disease spread or provide field-level intervention recommendations. To extend the system’s utility, future work should explore integrating disease spread prediction models and decision support for field interventions. Integrating the system with autonomous platforms such as drones and field robots could further enhance precision agriculture, offering comprehensive decision support and advancing the overall intelligence of the system.

The proposed multi-agent collaboration framework represents an innovative solution for plant disease detection, providing high accuracy, modularity, scalability, and robustness. By enabling independent yet coordinated agent operations, the system reduces processing latency, enhances real-time responsiveness, and maintains stability even in the face of agent failures. The inclusion of a visualization agent enhances interpretability and user-friendliness, lowering adoption barriers. Despite the challenges outlined, this collaborative paradigm lays the foundation for broader applications in intelligent and sustainable agriculture, with the potential to revolutionize the field of automated plant disease detection.

## 5 Conclusion

This study introduces Chat Demeter, an intelligent agent system for plant pest and disease management, powered by deep learning. represents a shift from traditional single recognition models to intelligent, interactive systems. The system integrates two core components: a Feature Fusion Transformer (FFT) module for image classification and a Retrieval-Augmented Generation (RAG) module for agricultural knowledge-based responses. The FFT module, which combines CNN and Transformer architectures, has demonstrated high accuracy in recognizing and classifying a wide range of plant diseases. In parallel, the RAG module enhances the system’s practical utility by incorporating retrieval mechanisms and agricultural knowledge bases, ensuring reliable responses to domain-specific queries. Comparative analysis with traditional plant disease recognition models reveals that Chat Demeter not only maintains high classification accuracy but also improves interactivity, allowing users to obtain diagnostic evidence and management recommendations more efficiently. While Chat Demeter shows promising results in both plant disease recognition and knowledge-based interaction, Additionally, Chat Demeter demonstrates significant advancements in domain adaptation, response accuracy, and interaction effectiveness within agricultural contexts. However, ongoing research will aim to refine these features further, particularly by improving the system’s robustness for a wider array of agricultural scenarios. Future work will focus on optimizing its performance further. Key areas of improvement include enhancing the system’s adaptability to newly emerging or rare diseases, exploring several directions to enhance the system’s capabilities. These advancements will help ensure the system’s scalability, practicality, and adaptability in diverse agricultural environments.

## Data Availability

The raw data supporting the conclusions of this article will be made available by the authors, without undue reservation.
